# Plasma from exercised rats administered to sedentary rats induces systemic and tissue inflammation

**DOI:** 10.14814/phy2.13087

**Published:** 2016-12-21

**Authors:** Georgios Goutianos, Aristidis S. Veskoukis, Aikaterini Tzioura, Vassilis Paschalis, Nikos V. Margaritelis, Konstantina Dipla, Andreas Zafeiridis, Ioannis S. Vrabas, Michalis G. Nikolaidis, Antonios Kyparos

**Affiliations:** ^1^ Department of Physical Education and Sports Science at SerresAristotle University of ThessalonikiSerresGreece; ^2^ Department of HematologyBlood BankGeneral Hospital of SerresSerresGreece; ^3^ Department of Physical Education and Sport ScienceNational and Kapodistrian University of AthensAthensGreece; ^4^ Department of Health SciencesSchool of SciencesEuropean University CyprusNicosiaCyprus; ^5^ Intensive Care Unit424 General Military Hospital of ThessalonikiThessalonikiGreece

**Keywords:** Blood, exercise, inflammation, muscle and adipose tissue

## Abstract

Recent studies have consistently supported the active role of blood in mediating biochemical and physiological tissue adaptations. However, no study has investigated the possible contribution of circulating factors in an exercise setting. The aim of the study was to investigate the role of circulating factors in exercise adaptations by chronically administering to sedentary animals blood plasma collected from acutely exercised animals. Phase 1: Blood plasma was collected from rats that swam to exhaustion and from sedentary rats. Phase 2: Other rats were divided into two groups (*n* = 20 per group): the first group involved rats that were injected intravenously with blood plasma originating from rats that previously swam to exhaustion, the second group consisted of rats that were injected intravenously with blood plasma originating from sedentary rats. Tail‐vein injections (2 mL/kg) were performed daily for 21 consecutive days. Inflammatory markers (C‐reactive protein, interleukins‐1α, 2, 6, 8, 10 and tumor necrosis factor‐a) were measured in blood plasma, muscle, and adipose tissue. Sedentary rats administered with plasma from exercised rats had significantly higher levels in all inflammatory markers measured in blood, skeletal muscle and adipose tissue, compared to the sedentary rats administered with resting plasma. Our data demonstrate that administration of “exercised” blood to sedentary rats induced inflammation in plasma, muscle and adipose tissue. Exercise adaptations are not solely due to intrinsic processes in muscle or adipose tissue. Blood factors also play a crucial role in mediating signals for tissue adaptations.

## Introduction

Blood tissue has been traditionally regarded as an inert body fluid, a kind of “sink” that passively accepts metabolic by‐products mainly generated by the contracting skeletal muscles and other tissues (Nikolaidis and Jamurtas 2009). However, blood also circulates a plethora of bioactive molecules (e.g., myokines, adipokines, and micro‐RNAs) that have been found to exert important biological effects on distant tissues (Pedersen and Febbraio 2012; Rowe et al. 2014). Some of the released molecules are transported in exosomes (protein‐lipid vesicles), which protect their bioactivity and enable their remote action (Tkach and Théry 2016). Therefore, the blood is the recipient of several secretomes, including those produced from its own cells (i.e., erythrocytes, leukocytes, and platelets), which constitutes a unique bioactive mixture. Recent high‐prolific studies introducing elegant experimental designs (e.g., cell‐tissue cultures incubated with plasma, incubations in mediums containing secretome of cells, parabiosis, plasma injection in living animals, isolated body part exercise) have consistently supported the active role of blood in mediating biochemical and physiological tissue adaptations (Conboy et al. 2005; Csiszar et al. 2009; Villeda et al. 2011, 2014; Catoire et al. 2012; Conti et al. 2012, 2013; Al‐Shanti et al. 2014; Stanford et al. 2015; West et al. 2015).

Inflammation is a physiological response of the immune system to harmful stimuli (e.g., pathogens) or physiological stress (e.g., exercise), and regulates fundamental biological processes (e.g., cell signaling). It is mediated by a variety of soluble factors, including a group of secreted polypeptides known as cytokines. They are divided in two main categories, namely pro‐inflammatory and anti‐inflammatory cytokines (Luster 1998). Pro‐inflammatory cytokines contribute to the regeneration of a healthy tissue as they trigger the degeneration and clearance of damaged or infected cells. The anti‐inflammatory cytokines control the pro‐inflammatory cytokine response. Therefore, it seems that there is an optimized balance between pro‐inflammatory and anti‐inflammatory response to cope with inflammation.

Skeletal muscle and adipose tissue are highly plastic tissues to exercise. Biological cues (e.g., inflammation) within these tissues are thought to drive their adaptive responses, directly contributing to improved physiological function. Importantly, blood supplies muscle and adipose tissues, allowing for potential communication with the systemic environment. Therefore, the possibility arises that peripheral systemic factors contribute to exercise adaptations in skeletal muscle and adipose tissue. Despite the fact that the active role of blood has been clearly revealed in aging, neurogenesis, glucose metabolism, and tissue regeneration (Conboy et al. 2005; Villeda et al. 2011, 2014; Al‐Shanti et al. 2014; Stanford et al. 2015), no study has investigated the possible contribution of circulating factors in an exercise setting. Considering that inflammation involves the coordinated communication of different cytokines produced from various tissues and immune cells via blood, plasma previously collected from acutely exercised rats was injected intravenously for 21 days to sedentary rats. We expected that the chronic administration of this presumably immune‐rich plasma, will mimic the acute exercise stimulus and will induce anti‐inflammatory responses in blood plasma, skeletal muscle, and adipose tissue of sedentary rats as if these rats were actually trained. A limitation of many studies in this field is that only circulating blood measurements have been used to investigate the immunological responses to exercise. Taking into account the importance of skeletal muscle and adipose tissue in production and adaptation to cytokines, we have included measurements in these tissues as well.

## Materials and Methods

### Animals

Adult male Wistar rats, weighing 380 ± 27 g were used in the study. The animals were housed under a 12 h light: 12 h dark cycle, controlled temperature (21–23°C) and controlled humidity (50–70%). Commercial rat chow and tap water were provided ad libitum. All procedures were in accordance with the European Union guidelines for the care and use of laboratory animals, as well as the “Principles of laboratory animal care” (NIH publication No. 86‐23, revised 1985). The project was reviewed and approved by the institutional review board and the appropriate state authority (#359888/3612).

### Study design

**Figure 1:**
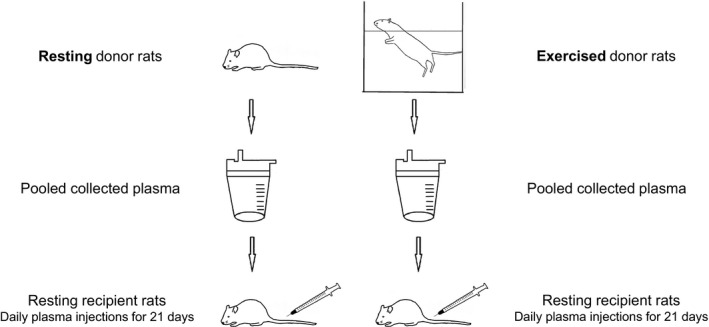
Study design.

Phase 1: Blood samples were collected: (1) from rats that swam to exhaustion, at cessation of exercise and (2) from sedentary rats, which were just placed in the swimming tanks filled with minimum amount of water. Whole blood samples were immediately centrifuged for separation of plasma from blood cells. Plasma samples were stored at −80°C. Subsequently, plasma samples from exercised or sedentary rats were pooled in two separate containers, homogenized (in each container), then separated into aliquots of 0.8 mL and stored at −80°C for use in phase 2 and for later analysis. Thus, in phase 1 there were essentially two kinds of pooled plasma, one collected from exercised rats, the other collected from resting rats and, therefore, they were treated as two single samples. Phase 2: Other rats were randomly divided into two groups (*n* = 20 animals per group): (1) the first group involved rats that were injected intravenously with blood plasma originating from rats that previously swam to exhaustion, (2) the second group consisted of rats that were injected intravenously with blood plasma originating from sedentary rats. Tail vein injections at the dose of 2 mL per kg body weight were performed daily for 21 consecutive days, using 1 mL insulin syringes. Twenty‐four hours after the last injection, rats of both groups were killed and blood, muscle, and adipose tissue samples were collected and stored at −80°C for later analysis. The study design is depicted in Figure 1.

### Swimming familiarization protocol

The rats were familiarized according to an appropriately modified protocol that was previously presented by our group (Veskoukis et al. 2008, 2012). The familiarization protocol lasted four consecutive days and the daily swimming duration was 10 min. On day 1, the rats swam free of load in order to avoid exacerbation of the anticipated stress caused by their first contact with water. On day 2, load equal to 5–10 g was adjusted at the base of their tails. On days 3 and 4, the additional load was 15 and 5 g, respectively. Then, the rats rested in their cages for 2 days before the graded swimming protocol until exhaustion.

### Swimming protocol

Rats swam individually in cylindrical tanks with a 1.2 m diameter and a height of 1.1 m. The depth of the water was 0.7 m to prevent rats from jumping out of the tank and from touching the bottom of the tanks with their tails. Water temperature was maintained between 33.5°C and 34.5°C. Rats performed a graded exercise test to exhaustion with gradually increasing loads adjusted at the base of their tails. Initial weight was 5 g for all rats. After the initial 15 min‐exercise, the load was increased to 10 g followed by 5 g increments every 5 min until exhaustion. An animal was considered to have reached exhaustion when it exhibited loss of coordinated movements and failure to return to the surface within 10 sec for three consecutive times (Veskoukis et al. 2008).

### Blood and tissue collection and preparation

Rats were deeply anesthetized by exposure to ether. The depth of anesthesia was assured by the constriction of the pupils as well as simple sensory tests, such as the absence of eye blinking when the eyelid was touched and the absence of foot withdrawal when the foot was pinched. Then, the thoracic cavity was opened. Whole blood was collected in tubes containing no additives (phase 1) or EDTA (phase 2) vacutainer tubes (BD Vacutainer Systems, Plymouth, U.K.), via cardiac puncture in the right ventricle, using a 10 mL syringe (Terumo, Tokyo, Japan). Whole blood samples were immediately centrifuged (1500*g*, 4°C, 10 min) for separation of plasma from blood cells. Plasma samples were stored at −80°C for later analysis. Immediately after blood sampling, vastus lateralis and epididymal fat pads were quickly excised, snap frozen in liquid nitrogen and stored at −80°C for subsequent analysis. In preparation for analysis, tissue samples were initially ground, using mortar and pestle under liquid nitrogen. A portion of the tissue powder was then homogenized with 10 mmol/L PBS (138 mmol/L NaCl, 2.7 mmol/L KCl, and 1 mmol/L EDTA, pH 7.4) and a cocktail of protein inhibitors (1 *μ*mol/L aprotinin, 100 *μ*mol/L leupeptin, and 1 mmol/L phenylmethylsulfonyl fluoride). The homogenate was vigorously vortexed and a brief sonication treatment on ice was applied. The homogenate was then centrifuged (12,000*g*, 4°C, 30 min) and the supernatant was collected and stored at −80°C.

### Biochemical analyses

All inflammatory markers (C‐reactive protein [CRP], interleukins‐[IL‐] 1α, 2, 6, 8, 10 and tumor necrosis factor‐a [TNF‐α]) were measured in plasma, muscle, and adipose tissue homogenates with Abbot Architect ci 8200 and Abbot Architect ci 16200 (Abbott Diagnostics, Abbot Park, IL) automated clinical analyzers. These analyzers are based on the method chemiluminescent magnetic immunoassay (CMIA). This method uses a chemiluminescent label that produces light when combined with a trigger reagent. The label of the devices is a patented acridinium derivative producing high light emission and thus high sensitivity as it is easier to measure a large amount of light. Furthermore, the CMIA method anchors antibodies via magnetic microparticles, a fact that makes this analytical method advantageous compared to ELISA assays. In particular, CMIA requires less dose of immunoreagents and time, while it provides a higher dose hook effect and bioactivity of immunoreagents as well as higher sensitivity, better reproducibility, and stability (Zhang et al. 2012; Liu et al. 2013).

### Statistical analysis

All variables were analyzed using two‐tailed unpaired Student's *t*‐tests (SPSS Inc., Chicago, IL; version 21). Data are presented as mean ± SD and percent changes. The significance level was set at *P* < 0.05. The pooled plasma samples (either exercised or resting) of phase 1 were treated as two single samples. As a result, no SD for these two pooled samples could be computed and no inferential statistics could be performed.

## Results

### Swimming protocol

Average swimming time was 28.92 ± 4.53 min and the average load was 2.62 ± 0.55% of the rats’ body weight.

### Inflammatory markers in plasma of resting or exercised rats administered to sedentary rats

**Figure 2:**
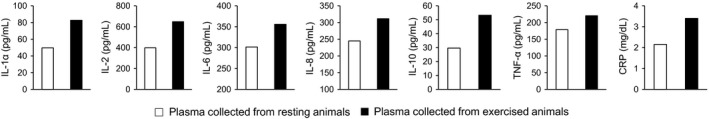
Inflammatory protein concentration in pooled plasma samples either collected from resting (open bars) or exercised (closed bars) animals. These plasma samples were administered in the two corresponding experimental groups in phase 2. Considering that the two kinds of pooled plasma were treated as two single samples (either exercised or resting), no inferential statistics could be performed.

The levels of cytokines in plasma collected from the swimming rats were higher compared to those in plasma from sedentary rats (IL‐1α [66%], IL‐2 [63%], IL‐6 [18%], IL‐8 [27%], IL‐10 [80%], TNF‐α [23%], CRP [58%]) (Fig. 2). This verifies the different cytokine and CRP composition of the two sets of plasma and the higher immune load injected to sedentary rats.

### Inflammatory markers in plasma, skeletal muscle and adipose tissue collected from sedentary rats following plasma administration from either resting or exercised animals

**Figure 3:**
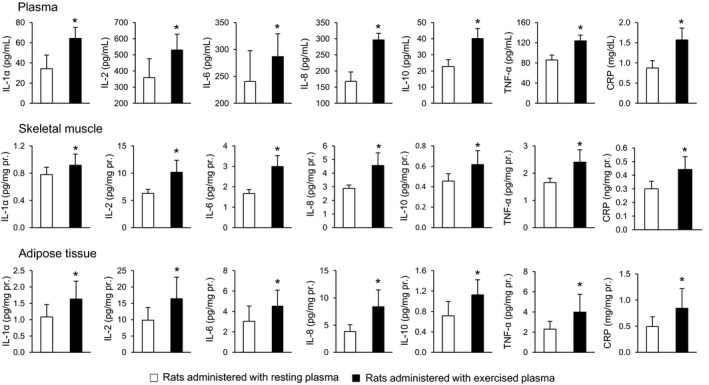
Inflammatory protein concentration in plasma, skeletal muscle, and adipose tissue of sedentary rats following plasma administration of either resting (open bars) or exercised (closed bars) animals. The asterisk denotes significant differences (*P* < 0.05).

Rats administered with plasma from exercised rats had significantly higher levels of all measured markers compared to the rats administered with resting plasma in circulating plasma (from 19% in IL‐6 to 88% in IL‐1α), in skeletal muscle (from 18% in IL‐1α to 79% in IL‐6), and in adipose tissue (from 50% in IL‐6 to 118% in IL‐8) (Fig. 3).

## Discussion

To our knowledge, this is the first study to investigate the role of circulating factors in exercise adaptations by chronically administering to sedentary animals blood plasma collected from acutely exercised animals. The principal finding of the study is that blood factors induced an inflammatory response, thus, affecting the inflammatory composition of tissues. Another finding of the present study was that the “exogenous” administration of inflammatory molecules did not induce the expected endogenous anti‐inflammatory response and adaptation. Our data did not confirm the hypothesis that the chronic injection of exercise‐derived inflammatory factors to sedentary rats will mimic the exercise stimulus per se and induce anti‐inflammatory responses and adaptations in blood plasma, skeletal muscle, and adipose tissue as if these rats were actually trained. On the contrary, we report that chronic plasma administration from exercised animals to sedentary animals induced an inflammatory state in blood and tissues of rats.

We found that the intravenous administration of plasma collected from exercised rats to nonexercised rats significantly increased the concentration of the pro‐ and anti‐inflammatory markers (IL‐1α, IL‐2, IL‐6, IL‐8, IL‐10, TNF‐α, CRP) measured in plasma, muscle, and adipose tissue of sedentary rats. This finding is in line with previous in vitro and in vivo studies demonstrating that circulating factors can affect the biochemical milieu and the physiological characteristics of distant tissues (i.e. Conboy et al. 2005; Csiszar et al. 2009; Villeda et al. 2011, 2014; Catoire et al. 2012; Conti et al. 2012, 2013; Al‐Shanti et al. 2014; West et al. 2015). Although our findings confirm the “signaling” role of blood constituents, surprisingly, the injected “exercised” plasma triggered an inflammatory response in blood plasma, vastus lateralis, and adipose tissue. This is difficult to explain as we were expecting that daily exposure of muscle and adipose tissue to altered levels of circulating cytokines (mimicking the acute episodes of inflammation induced after endurance exercise) could eventually mitigate inflammation in blood and tissues.

As expected, the plasma collected from acutely exercised rats had significantly higher concentration in cytokines and CRP compared to plasma collected from sedentary rats. It is noteworthy that the inflammatory response of the sedentary rats that received the “exercised” plasma was achieved with a very small dose of inflammatory stressors, suggesting the high bioactivity of these factors. Using the formula of Lee and Blaufox (1985), it is estimated that the blood volume of a rat weighing 390 g is 24.17 mL. The injected plasma dose for an animal weighing 390 g was 0.78 mL (2 mL/kg body weight), which denotes that the injected dose constituted 3.23% of the total blood volume. For example, TNF‐α concentration in the “exercised” plasma was 23.2% higher compared to plasma collected from sedentary rats. This additional 23.2% was diluted 31 times (100/3.23 = 31), thus, the injected exercise plasma could account for only 0.75% (23.2%/31 = 0.75%) of changes in plasma after administration. However, the actual increase of TNF‐α in sedentary rats injected with “exercised” plasma was 44.2%, revealing a significant endogenous production of TNF‐α. Regarding skeletal muscle, the theoretical 0.75% change of TNF‐α in plasma is then dispersed to skeletal muscles, which constitute ~40% of total body weight and, thus, could account for only 0.3% increase in vastus lateralis. The actual increase in TNF‐α of vastus lateralis was 45.5% demonstrating a large endogenous production of TNF‐α in skeletal muscle as well.

Therefore, an emerging question is how administration of “exercised” plasma in sedentary rats evoked an endogenous production of cytokines and CRP. It is apparently very difficult to provide mechanistic explanations for our findings. It is likely that blood cells and tissues “sensed” the homeostatic perturbation evoked by the administered “exercised plasma” as an “assault” and responded by increasing secretion of inflammatory proteins. Successful inflammatory response is followed by the resolution phase that restores homeostasis. However, the fact that inflammation was induced by a nonphysiological stimulus (i.e., exogenous plasma injection) may have resulted in incompatible homeostatic processes, which, in turn, led the system to become “locked” in a state of a chronic inflammation that failed to be resolved (Kotas and Medzhitov 2015). In our study, the inflammatory factors were administered for 21 consecutive days. It might be that the “recovery” elapsed time among doses was not sufficient for the system to restore its inflammatory homeostasis. Our findings are similar to those of Krishnamurthy et al. (2012) where infusion of 100 *μ*L of plasma, derived from heat shock factor protein 1 knockout mice, was nonproportional to the injected dose increase of heat shock protein 25 levels, possibly by activating circulating macrophages.

A limitation of the present study may be the fact that there was no comparison of plasma/tissue inflammation from exercised rats with a sedentary control group, which did not receive any plasma. In that case, perhaps the effects of plasma itself would become a bit clearer. However, the “sedentary plasma” (from sedentary rats) administered to the sedentary group may also be considered as a control. In a perspective, the present study may initiate further studies in this field, possibly involving different doses and administration periods, longer follow up and a comparison between different exercise protocols, with the aim to characterize the long‐term adaptive response induced by the exercise.

## Conclusion

This study aimed to combine the strengths of a physiologically derived intervention (i.e., exercise‐induced biochemical milieu) with the experimental advantages of an ex vivo model (i.e., plasma injection). Using this ex vivo – in vivo approach, our data demonstrate that intravenous administration of “exercised” blood to sedentary rats induced inflammation in plasma, muscle, and adipose tissue. It is important to consider that a miniscule injection dose was able to induce large adaptations. Our data strongly support the view that exercise adaptations are not solely a muscle or adipose tissue intrinsic processes, but that blood factors also play a crucial role in mediating signals for tissue adaptive responses. A better understanding of how circulatory factors affect distant tissues and identification of specific molecules are of paramount importance, as they will possibly lead to development of interventions able to reproduce (at least partially) exercise/training effects. Such strategies could be advantageous for people who are unable to exercise.

## Conflict of Interest

None declared.
